# Cuts through the manifold of molecular H_2_O potential energy surfaces in liquid water at ambient conditions

**DOI:** 10.1073/pnas.2118101119

**Published:** 2022-07-05

**Authors:** Annette Pietzsch, Johannes Niskanen, Vinicius Vaz da Cruz, Robby Büchner, Sebastian Eckert, Mattis Fondell, Raphael M. Jay, Xingye Lu, Daniel McNally, Thorsten Schmitt, Alexander Föhlisch

**Affiliations:** ^a^Institute Methods and Instrumentation for Synchrotron Radiation Research, Helmholtz Center Berlin for Materials and Energy, 12489 Berlin, Germany;; ^b^Institute of Physics and Astronomy, University of Potsdam, 14476 Potsdam, Germany;; ^c^Photon Science Division, Swiss Light Source, Paul Scherrer Institut, CH-5232 Villigen PSI, Switzerland

**Keywords:** water, potential energy surface, RIXS

## Abstract

The fluctuating hydrogen bridge bonded network of liquid water at ambient conditions entails a varied ensemble of the underlying constituting H_2_O molecular moieties. This is mirrored in a manifold of the H_2_O molecular potentials. Subnatural line width resonant inelastic X-ray scattering allowed us to quantify the manifold of molecular potential energy surfaces along the H_2_O symmetric normal mode and the local asymmetric O–H bond coordinate up to 1 and 1.5 Å, respectively. The comparison of the single H_2_O molecular potentials and spectroscopic signatures with the ambient conditions liquid phase H_2_O molecular potentials is done on various levels. In the gas phase, first principles, Morse potentials, and stepwise harmonic potential reconstruction have been employed and benchmarked. In the liquid phase the determination of the potential energy manifold along the local asymmetric O–H bond coordinate from resonant inelastic X-ray scattering via the bound state oxygen 1*s* to $4a_{1}$ resonance is treated within these frameworks. The potential energy surface manifold along the symmetric stretch from resonant inelastic X-ray scattering via the oxygen 1*s* to $2b_{2}$ resonance is based on stepwise harmonic reconstruction. We find in liquid water at ambient conditions H_2_O molecular potentials ranging from the weak interaction limit to strongly distorted potentials which are put into perspective to established parameters, i.e., intermolecular O–H, H–H, and O–O correlation lengths from neutron scattering.

## Significance

Liquid water at ambient conditions is ubiquitous in chemistry and biology as well as in technology, energy, and atmospheric processes. Since parts of the phase diagram of water are unsettled—most notably the supercooled liquid homogeneous nucleation region—repercussions thereof on our molecular-level understanding for even the common ambient conditions remain. Breathtaking advances in X-ray–based approaches over the last decade give us now the tools to derive molecular potential energy surfaces as a quantitative view on the molecular manifold within the fluctuating hydrogen bonding network. With selective cuts along the local asymmetric O–H bond coordinate and the symmetric normal mode excitations an experimental foundation to benchmark competing molecular-level models of water has been achieved.

Although all properties of the H_2_O molecule itself are exceedingly well described by experimental and computational approaches, the condensed phases of ice (1) and liquid water (2) harbor unresolved aspects of emergent properties. In particular, the liquid phase at ambient conditions could be influenced by the extrapolation of the divergence of thermodynamic response functions in the supercooled region (3, 4). The ground state potential energy surface (PES) of the H_2_O molecule is fully described by the three molecular normal coordinates of symmetric and antisymmetric stretch and the H–O–H bend. With subnatural line width resonant inelastic X-ray scattering (RIXS) and ab initio theory (5), cuts through the molecular ground state PES have been achieved in the gas phase water molecule, most notably, along the normal symmetric mode and the local asymmetric O–H bond coordinate (6). Also, mode coupling and interference aspects have been fully described (7, 8). Finally, the sensitivity of this approach to hydrogen bridge bonding has been established in an azeotrope mixture (9).

In the condensed phases of water, the hydrogen bridge bonding network leads not only to modifications to the local molecular potentials, but also creates properties on longer correlation length scales, most notably the various crystalline and amorphous solid phases. For the fluctuating hydrogen bridge bonded network of the equilibrium and nonequilibrium liquid phases, ongoing debate touches the question to what extent emergent phenomena and resulting nanometer correlations play a role at ambient conditions. This debate hinges on the question whether liquid water at ambient condition might inherit collective properties stemming from the critical fluctuation physics of the purported low- and high-density nonequilibrium liquid phases in the supercooled homogeneous nucleation region.

The underlying conceptual frameworks are the following: H_2_O ice, with its local fourfold, tetrahedral coordination shares the general physics of tetrahedral coordinated crystals and melting such as silicon or germanium (10, 11). These systems exhibit liquid phase density anomalies from a volume compacted thermodynamic stable high-density liquid phase which is accompanied by a transient supercooled low-density liquid phase. In the phase diagram this leads to a phase separation line, critical fluctuations, a critical point, and the Widom line beyond that critical point. Since water and ice fulfill these conditions, the liquid–liquid critical point scenario has been proposed (4, 12, 13). In this, the amorphous and crystalline ice phases extend into the supercooled homogeneous nucleation region of water as phase separated nonequilibrium low- and high-density liquid phases. These mandate the existence of the second critical point, which in turn could lead to a Widom line and critical fluctuation phenomena. Conceptually deviating critical point–free or continuous scenarios have equally been fielded (14–16).

Liquid water in equilibrium at ambient conditions resides thermodynamically far above this region of the phase diagram and the energy scales of critical fluctuations around the purported phase separation line. Nevertheless, prevalence of continuous distribution at ambient conditions could be accompanied by some degree of rapidly fluctuating correlations. Thus, the manifold of molecular ground state potentials constitute the statistical distribution leading to the ensemble average of all molecular moieties in the fluctuating hydrogen bridge bonding network of liquid water at ambient conditions. Quantitative detection and analysis of X-ray spectroscopy yielded a mean value of 1.74 ± 2.1% donated and accepted hydrogen bonds per molecule in liquid water at ambient conditions (17).

In this work, we access and quantify the manifold of electronic ground state molecular PESs along the local asymmetric O–H bond coordinate (asymmetric coordinate) and the symmetric normal mode (symmetric coordinate) within the molecular ensemble average of liquid water at ambient conditions in direct comparison to the PES of the isolated H_2_O molecule. No prevalence of distinct structurally driven coordination scenarios is observed, but a coexistence of a broad manifold of PESs prevails.

## Results and Discussion

**Fig. 1.**
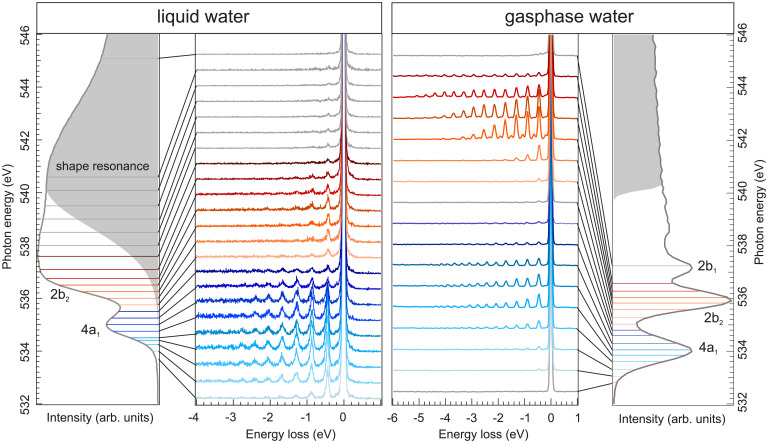
Vibrationally resolved RIXS spectra of liquid and gas phase water, excited across the O 1*s* absorption resonance. The first and fourth panels show the respective absorption spectra with the IP marked in gray. The blue and red lines are the measured excitation energies for the 4*a*_1_ prepeak and 2*b*_2_ main resonance, respectively. Other excitation energies are in gray.

In Fig. 1, vibrationally resolved RIXS of liquid and gas phase water excited across the O 1s absorption resonance is shown together with the soft X-ray absorption spectrum of liquid water and the electron energy loss spectrum of gas phase water from ref. 18.

Comparing the absorption spectra of liquid and gas phase water, we observe for the isolated molecule in gas phase water three distinct absorption resonances, $4a_{1},\;2b_{2}$, and $2b_{1}$. The ionization potential (IP) is well separated from the resonances, and its width is only defined by the lifetime of the core excited intermediate state [160 meV (19)]. The liquid, on the other hand, shows broad and overlapping resonances with the $4a_{1}$ being the lowest state, followed by the $2b_{2}$. Due to the hydrogen bond network, the IP in liquid water (1.57 eV at 538.5 eV) is shifted lower and broadened compared to the gas phase, representing the ensemble average over many chemically different molecular structures, partly overlapping and mixing with the molecular $2b_{1}$. Liquid water also has well above IP a distinct shape resonance at 541 eV from the second coordination shell (17).

The vibrational progressions in the RIXS spectra (second and third panels in Fig. 1) show striking differences between the liquid and gas phase. The gas phase has distinct long vibrational progressions, caused by selective excitation of the local asymmetric O–H bond coordinate, the symmetric stretch, and H–O–H bend for each X-ray absorption resonance $4a_{1},\;2b_{2}$, and $2b_{1}$ (6). The vibrational peak width depends only on the experimental resolution and is thus of symmetric shape. For the liquid, similar but shorter progressions are found. Here the intermolecular interactions introduced by the hydrogen bond network lead to increased width and asymmetry for higher overtones as well as the emergence of a continuous background for excitation around the $4a_{1}$ (20).

**Fig. 2.**
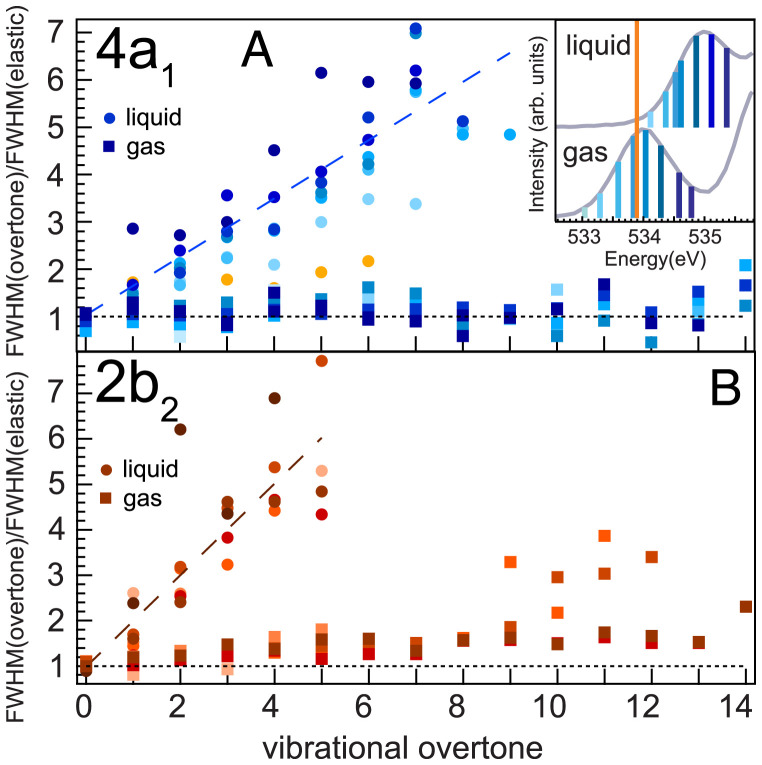
Width of vibrational overtones normalized to the incident photon bandwidth (elastic line width) for excitation across (*A*) $4a_{1}$ and (*B*) $2b_{2}$. Gas phase overtones experience no broadening over the incident photon bandwidth. The liquid phase overtones increase width linearly with higher vibrational excitation due to the hydrogen bridge bonding interaction. In the liquid, the rate of broadening increases from below threshold (light blue) excitation across the $4a_{1}$ X-ray absorption resonance (dark blue). A resemblance with gas phase properties is observed for strongly detuned excitation in the liquid phase (orange).

In Fig. 2, let us quantify and summarize the overtones and their width for all excitation energies across the $4a_{1}$ leading to excitation of the local asymmetric O–H bond coordinate. As many as 14 vibrational overtones have been resolved in the gas phase, in contrast to only 9 in the liquid. In each case, the vibrational spacing carries ground state potential energy information (9, 20, 21). The incident photon energy bandwidth of subnatural linewidth RIXS is conserved in both the gas phase and liquid phase best visible in the elastic line without vibrational excitation. Thus, normalizing the width of higher vibrational modes yields for each vibrational level the system-dependent broadening as the ratio of the full width at half maximum (FWHM): FWHM (overtone)/FWHM(elastic).

As seen in Fig. 2*A*, the free, noninteracting molecule has a constant ratio of 1, independent of the vibrational level, for the asymmetric coordinate excited via the $4a_{1}$. The symmetric coordinate excitation (Fig. 2*B*) via the $2b_{2}$ also stays at constant ratio 1, but at high vibrational overtones, coupling between the symmetric and H–O–H bend normal modes starts to influence the peak shape (8). In the liquid, both the asymmetric and the symmetric coordinate show a broadening increase with overtone number not present in the gas phase. The broadening of higher overtones in the liquid results from an overlap of PES from different local environments leading to many close-lying energy eigenvalues. These different local environments are the result of intermolecular interactions (e.g., H bonds) affecting the potential shapes. The farther out nuclear motion is driven by core excitation, the more sensitive the reconstructed PES is to these local potential changes by neighboring molecules. In particular, excitation into the symmetric normal mode via $2b_{2}$ broadens more than excitation into the local asymmetric coordinate via the $4a_{1}$. This indicates that along the symmetric stretch, stronger intermolecular interactions apply than for the asymmetric coordinate. As we will see later in this section, this gradual broadening relates directly to the manifold of ground state PESs in liquid water.

We also note that exciting below the maximum of the $4a_{1}$ resonance leads with larger photon energy detuning to a reduced vibrational overtone broadening in the liquid (Fig. 2*A*, detuning dark to light blue). For strong detuning the vibrational peak shapes gradually tend toward the limit of the free molecule case without reaching it in the liquid phase. To highlight this, we mark the nominal gas phase X-ray absorption energy of 533.80 eV with an orange line in the X-ray absorption in Fig. 2 *A*, *Inset*, and orange circles in the ratio of vibrational overtone broadening.

**Fig. 3.**
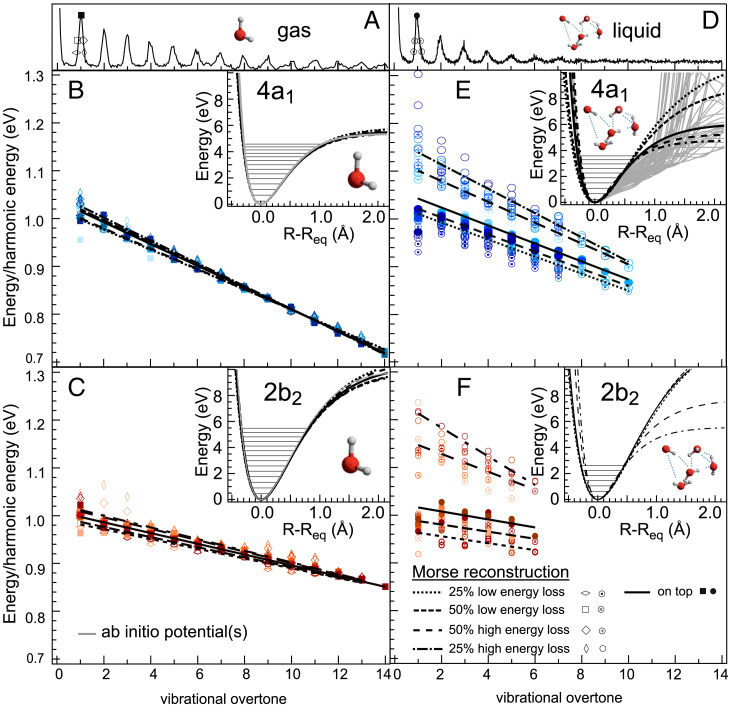
Vibrational progressions and peak energies relative to the energy of a harmonic potential E*_m_*/E*_h_* of (*A*–*C*) gas phase and (*D*–*F*) liquid water excited from strongly detuned (light blue) across the 4*a*_1_ resonance (dark blue) as well as across the 2*b*_2_ resonance (light red to dark red). For each excitation energy, the different markers represent the energy position of the vibrational peak taken at the following positions: on top of the peak (filled markers) or at 50 or 25% peak intensity at the low–and high–energy loss sides (see *A* and *D* for respective energy positions). For gas phase, all E*_m_*/E*_h_* exhibit linearity, indicating the validity of the Morse reconstruction approach. The hydrogen bond network in liquid water leads to a multitude of different PESs in ab initio molecular dynamics calculations on the BLYP level (gray curves, *Inset* in *E*; taken from ref. 20), which are not reflected in the Morse reconstruction: E*_m_*/E*_h_* is much wider spread for liquid, showing clear deviation from linearity.

We can now gain information about the liquid water ground state PES by extraction of its shape from the vibrational progressions. For isolated molecules, a Morse fit procedure can be employed, and in the case of gas phase water we have shown that highly accurate one-dimensional cuts through the ground state PESs are extracted (6, 7). To visualize the validity of this approach, we plot the ratio of the measured vibrational peak energy and the energy of a harmonic potential E*_m_*/E*_h_* in dependence of the vibrational overtone. In Fig. 3, E*_m_*/E*_h_* is shown for all excitation energies across the $4a_{1}$ (Fig. 3*B*) and $2b_{2}$ (Fig. 3*C*) resonances in gas phase water. The clear linear dependence on the overtone number implies that the measured energies E*_m_* follow a Morse potential (*SI Appendix*). The PESs extracted from the experimental data using Morse reconstruction are shown in the Fig. 3 *B* and *C*, *Insets* together with the ab initio calculated PES using the scalar relativistic restricted–active space self-consistent field method (22) followed by second-order perturbation theory method (23) from ref. 6. All these potential curves coincide nicely, supporting the Morse ansatz describing the gas phase molecule along the symmetric and local asymmetric O–H bond stretch coordinate.

In the case of liquid water, however, this is not the case. The ratios E*_m_*/E*_h_* plotted in Fig. 3 *E* and *F* show a much larger spread of E*_m_*/E*_h_* for different excitation energies as well as for different energy positions on the vibrational peak, the slopes of the Morse reconstruction differing clearly. Comparing the Morse extracted PESs in Fig. 3 *E*, *Inset* with ab initio molecular dynamics calculations on the Becke–Lee–Yang–Parr (BLYP) functional level (24, 25) of the manifold of PESs that reflect the different geometrical molecular structures in the hydrogen bond network of liquid water on the $4a_{1}$ resonance from ref. 20, we find that the Morse reconstruction gives the steep limit of these set of curves, i.e., the single-molecule limit with weak H-bonding (Fig. 3*F*). This is also true in the case of $2b_{2}$ symmetric stretch excitation, where due to the delocalized nature of the excited state, ab initio calculations are not possible.

The wider potential surfaces where H bonds play a significant role are not reproduced by the Morse reconstruction. In order to take a turn on reconstructing even those, we propose the approach of stepwise harmonic reconstruction where we approximate the local width of the PES at a certain vibrational overtone energy by the width of a harmonic potential of that overtone energy. Using the gas phase Morse potential PES for $R\leq R_{eq}$ as the short bonding flank, the reconstructed potential widths give the long bonding flank (*SI Appendix*). This approach of stepwise harmonic reconstruction allows us to derive now the full manifold of potential cuts straight out of the experimental values.

**Fig. 4.**
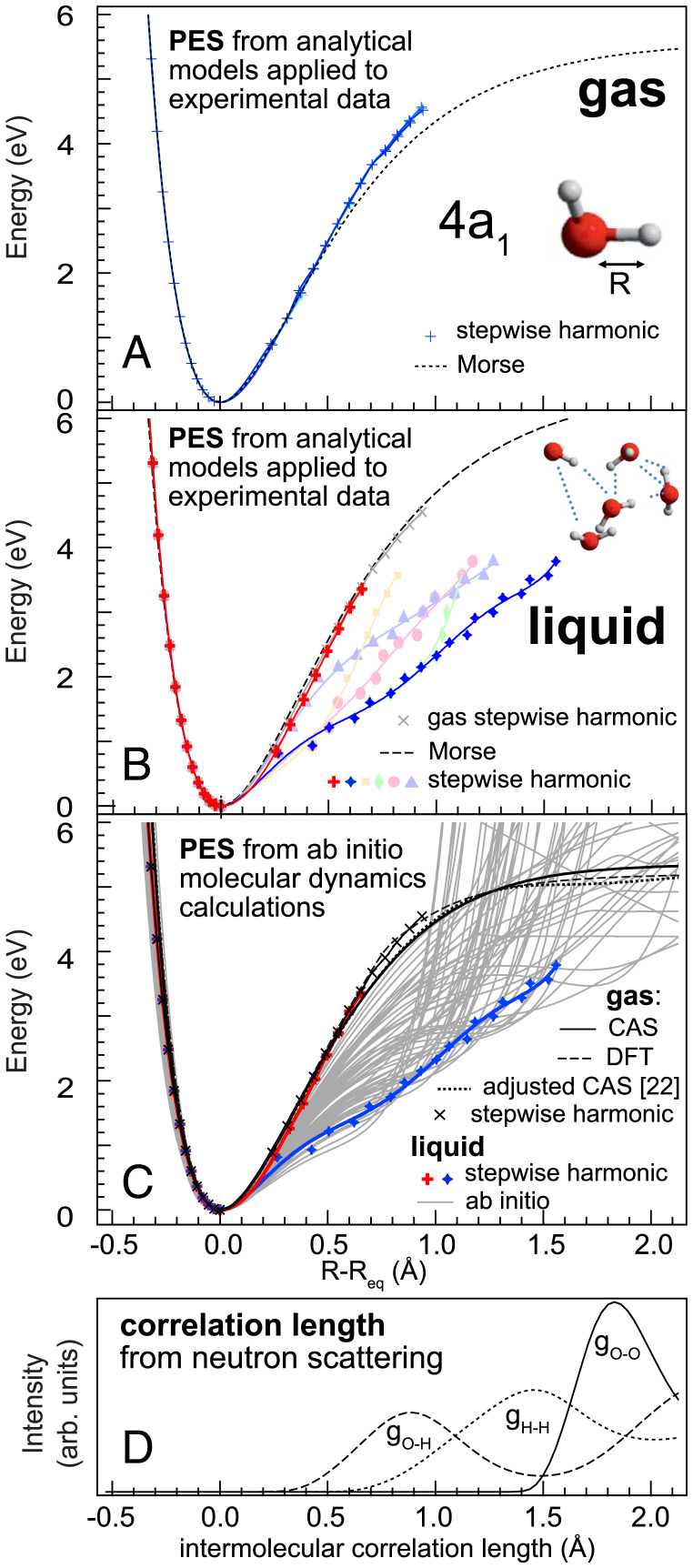
Reconstruction of one-dimensional PES cuts of liquid water along one O–H bond using analytical Morse and stepwise harmonic reconstruction applied to the experimental data of (*A*) gas phase and (*B*) liquid water. To illustrate the multitude of possible potential shapes to be reconstructed for liquid, the steepest (red) and shallowest (blue) potentials are shown together with a number of representative intermediate potentials (pale colors); the detailed parameters of these are given in *SI Appendix*. (*C*) Comparison of reconstructed liquid PES to ab initio molecular dynamics calculations (gray) from ref. 20. While the Morse reconstruction only describes the steep limit of the PESs (black solid line), the stepwise harmonic reconstruction allows us to capture even the non–Morse shaped PESs with strong hydrogen bonding character (blue lines and markers). (*D*) Intermolecular radial distribution functions of liquid water based on neutron scattering (from ref. 26), showing correlation between *g_OO_* and the sudden increase of most of the ab initio potentials in *C* resulting from the presence of neighboring molecules.

To verify this procedure, we apply the stepwise harmonic reconstruction to gas phase water on the $4a_{1}$ and obtain the blue curves in Fig. 4*A*. They match the Morse reconstruction and the ab initio calculations for bond elongations up to $R-R_{eq}$ = 1 Å exceedingly well (see also Fig. 3 *B*, *Inset*) and have only a tiny offset to even higher bond elongation $R-R_{eq}$. Fig. 4*B* turns to liquid water and finds also there the limit of a quasi-free molecular limit as seen by the potential given by the red crosses from stepwise harmonic reconstruction. This curve coincides with both the molecular Morse reconstruction (Fig. 4*B*) and the steepest potential curve from previous ab initio calculations (Fig. 4*C*).

We know from our previous work on liquid water (20) with ab initio molecular dynamics calculations that the partial densities of the vibrational states overlap for higher overtones, thus allowing more than one (i.e., one or two) energy eigenvalues per experimental vibrational peak for all vibrational peaks above the first overtone (20). Adapting the stepwise harmonic reconstruction to this fact, we obtain the results shown with colored markers in Fig. 4*B* for distinct sets of eigenvalue combinations to illustrate the possibility to reconstruct a multitude of different potential shapes (for details, see *SI Appendix*). In sum, Fig. 4*B* shows for the local asymmetric coordinate of the $4a_{1}$ excitation that stepwise harmonic reconstruction from purely experimental values allows us to derive the manifold of PESs to high precision as compared to ab initio computational models.

However, the stepwise harmonic reconstruction has a systematic error and is not self-consistent: the energy eigenvalues of the reconstructed potential are not the same as the measured input energies, even though they do not differ by much. However, even though we cannot obtain quantitative information on the potentials, we gain access to qualitative potential shapes along different coordinates. A further clear benefit is that we can also reconstruct nondissociating potentials and potentials with odd shapes.

Since the PESs along the local asymmetric coordinate reach far out, it is instructive to compare these elongated bond situations to the radial distribution functions present in liquid water obtained by neutron scattering (26), plotted in Fig. 4*D*. Here curve *g_OO_* corresponds to distance distribution of the oxygen atom of a neighboring molecule, and *g_OH_* and *g_HH_* correspond to those of the intermolecular oxygen–hydrogen and hydrogen–hydrogen pairs, respectively. We observe how these length scales are at values of significant change in the PES manifold of liquid water.

**Fig. 5.**
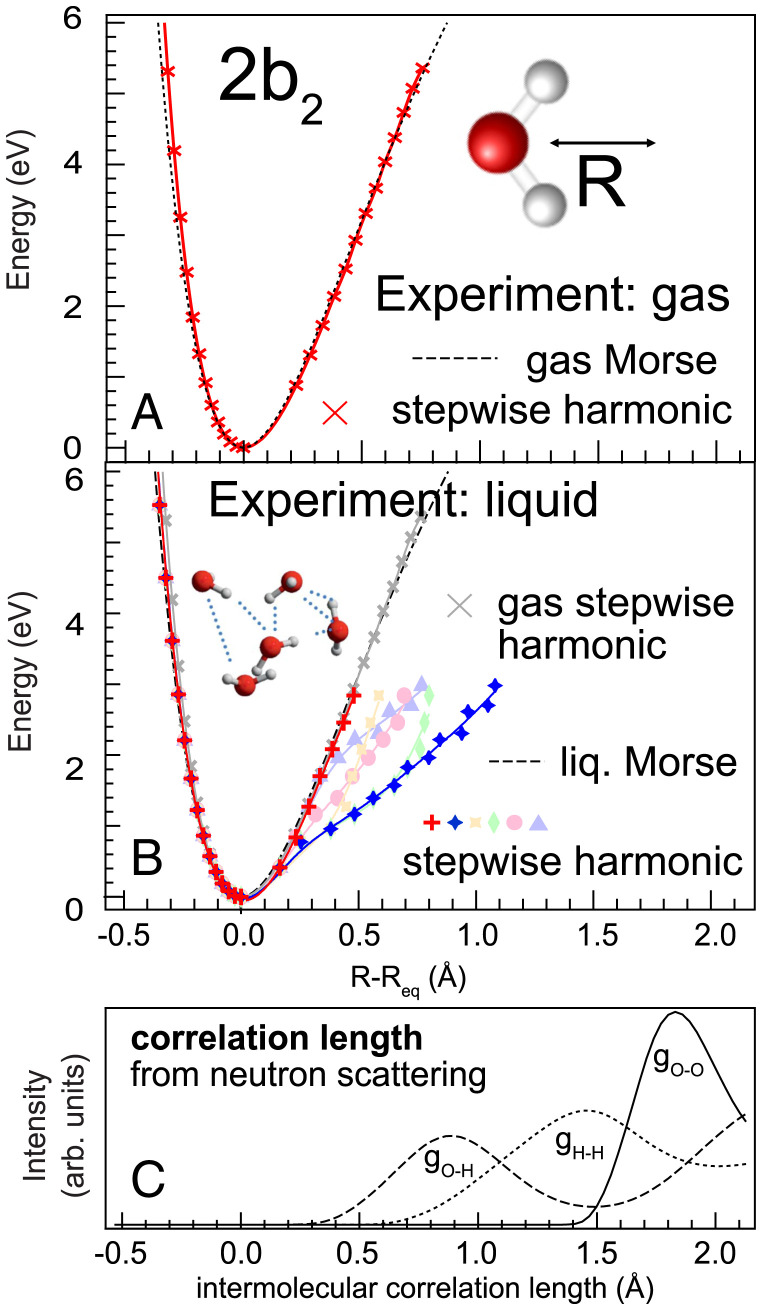
PES for water excited at the $2b_{2}$ main resonance, yielding a cut through the ground state PES along the diagonal between the two OH bonds for (*A*) gas phase and (*B*) liquid water reconstructed using the stepwise harmonic model. The liquid exhibits an ensemble of PES from different geometrical structures: the steepest (red) and shallowest (blue) potentials are shown together with a number of representative intermediate potentials (pale colors); the detailed parameters of these are given in *SI Appendix*. (*C*) Intermolecular radial distribution functions *g_OH_*, *g_HH_*, and *g_OO_* of liquid water based on neutron scattering (from ref. 26).

Having described the $4a_{1}$ (in Fig. 2) with its local asymmetric coordinate, we also want to describe the PES of the symmetric coordinate at the $2b_{2}$ (in Fig. 5). The overlapping continuum states at the $2b_{2}$ make a first principles RIXS computation problematic, and we thus utilize our established stepwise harmonic reconstruction for both the gas and liquid phase of water. For both the $4a_{1}$ and the $2b_{2}$ excitation the number of vibrational overtones is similar in the gas phase (Fig. 3). In the liquid, the number of resolved peaks corresponding to excited overtones is smaller in the $2b_{2}$ compared to the $4a_{1}$ (thus showing it to be unlikely that both hydrogens of the molecule point off the neighboring water molecules) and without a continuous background that could mask additional overlaying vibrational states (Fig. 3).

In Fig. 5*A* we see how the Morse and stepwise harmonic reconstruction coincide for the gas phase exceedingly well. For the liquid in Fig. 5*B*, we obtain a set of PESs along the symmetric stretch coordinate that are present in the ensemble average of liquid water up to $R-R_{eq}=1$ Å symmetric stretch elongation. In this range the *g_OH_* and to a lesser extent the *g_HH_* correlation length scales reside and influence the molecular ensemble PES.

## Conclusion and Outlook

For the water molecule in the gas phase and the liquid phase we have determined the manifold of ground state PES cuts along the symmetric and local asymmetric stretch coordinates up to $R-R_{eq}=1.5$ Å bond elongation. These results are based on the detailed analysis of vibrational progressions and peak shapes of subnatural line width oxygen K-edge RIXS with ab initio, Morse, and stepwise harmonic reconstruction. The latter is a powerful approach to extract PESs from experimental high-resolution RIXS spectra that we have benchmarked for the local asymmetric O–H bond coordinate to ab initio and Morse approaches. Within the manifold of the molecular H_2_O PESs, we find next to the strongly distorted situations also the limit case of weak hydrogen bonding of an almost gas-like H_2_O along the local asymmetric coordinate. No prevalence of distinct structurally driven coordination scenarios is observed, but a coexistence of a broad manifold of PESs prevails. All PESs of the manifold can be related to the established radial distribution functions $g_{OH},$ $g_{HH},$ and *g_OO_* of liquid water based on neutron scattering.

## Materials and Methods

### Experimental methods.

The spectra were measured using the SAXES spectrometer (27) of the ADRESS beamline (28) at the Swiss Light Source of the Paul Scherrer Institut (PSI). A liquid flow cell was used with a 150 nm Si_3_N_4_ membrane with 10 nm Au coating separating the vacuum from the sample. The scattered photons were detected with an angle of 90^∘^ from the incoming photons with a combined resolution of 75 meV for gas phase water and 45 meV for liquid water. The energy calibration of the excitation energy was done using the CO absorption maximum, while the RIXS energy calibration utilized the O_2_ gas vibrational progression (21). To prevent breaking of the membrane under irradiation, the cell was moved every 10 min. The individual spectra were measured for 5 min intervals, and the scans were shifted to the same energy scale using the position of the elastic line as an indicator.

The vibrational overtones in the RIXS spectra were fitted by Voigt profiles with free width and an additional free parameter to account for asymmetric peak shapes to get the best possible fit. To obtain the overall peak width, the fit results were then evaluated in the following way: the maximum intensity of each fitted overtone was taken as the peak energy position, the peak width (FWHM) is the sum of the two half-widths at half maximum (HWHM ) defined as the energy difference of the energy of the peak maximum intensity to that at half intensity separately for left and right side, FWHM = HWHM*_left_* + HWHM*_right_*. The error of the width determination is represented by the spread of the values for the free, noninteracting molecule.

The experimental spectra of the prepeak region of liquid water have been taken from ref. 20, the spectra from gas phase water have been taken from ref. 8, and the other spectra were previously unpublished.

## Supplementary Material

Supplementary File

## Data Availability

All study data are included in the article and/or supporting information.
