# Supplementary material for: Cuts through the manifold of molecular H2O potential energy surfaces in liquid water at ambient conditions
Source: Proc Natl Acad Sci U S A. 2022 Jul 5;119(28):e2118101119. doi: 10.1073/pnas.2118101119 (PMC9282235; doi:10.1073/pnas.2118101119)
Supplement: Supplementary File [file pnas.2118101119.sapp.pdf]

Supplementary Information for

Cuts through the manifold of molecular H_2O potential energy surfaces in liquid water at ambient conditions

Annette Pietzsch, Johannes Niskanen, Vinicius Vaz da Cruz, Robby Büchner, Sebastian Eckert, Mattis Fondell, Raphael M. Jay, Xingye Lu, Daniel McNally, Thorsten Schmitt, and Alexander Föhlisch

Annette Pietzsch and Alexander Föhlisch.
E-mail: annette.pietzsch@helmholtz-berlin.de, alexander.foehlisch@helmholtz-berlin.de

This PDF file includes:

Supplementary text
Figs. S1 to S4
Tables S1 to S2
References for SI reference citations

Supporting Information Text

Ratio of the measured vibrational peak energy and the energy of a harmonic potential E_M/E_h

The energy E_M for the overtone n in a Morse potential is given by:

$$E_M = h\nu(n+\frac{1}{2}) - \frac{h^2\nu^2}{4D}(n+\frac{1}{2})^2 \quad [1]$$

where h is the Planck constant, ν the frequency, D the dissociation energy. The corresponding energy E_h in a harmonic potential for the nth overtone is given by

$$E_h = -h\nu(n+\frac{1}{2}) \quad [2]$$

The ratio E_M/E_h of these two energies gives than a linear dependency on the overtone number:

$$\frac{E_M}{E_h} = \frac{h\nu(n+\frac{1}{2}) - \frac{h^2\nu^2}{4D}(n+\frac{1}{2})^2}{h\nu(n+\frac{1}{2})} = 1 - \frac{h\nu}{4D}(n+\frac{1}{2}) \quad [3]$$

Stepwise harmonic reconstruction

The stepwise harmonic reconstruction bases upon the following concept: For each vibrational overtone energy, the local width of the PES is approximated separately by the width of a harmonic potential of that overtone energy. To obtain the corresponding relative bond elongation, the limit of short bond is defined by the short bonding flank of the gasphase potential. From that limit, the approximated width from the harmonic potential gives the high relative limit of bond elongation.

For the extraction of the potential energy surface, we start with the part of the measured gas phase potential with $R < R_{eq}$ as the low limit for bond length. In this region the water potential is dominated by the coulombic contribution which is similar for gasphase and liquid. Alternatively, one could also use that part of a calculated potential from density functional theory (DFT) or restricted active space (RAS) since the multi-configurational terms can be neglected in this range, see the comparison in Fig. S2 for gasphase water at the 4a1.

The section of the potential with $R > R_{eq}$ (the high limit of bond length) is reconstructed using the experimental vibrational energies E_n which are obtained by fitting the RIXS spectra with a number of Gaussian peaks with free width to accommodate for the increasing width of the higher vibrational overtones due to excitation of a multitude of different molecular configurations.

Starting with the gas phase potential for $R \leq R_{eq}$, the potential is now being expanded towards $R > R_{eq}$ by calculating for each vibrational overtone n and the corresponding experimental energy E_n the harmonic potential V that is defined as

$$V(r) = \frac{1}{2}\omega^2 m_{red} r^2 \quad [4]$$

where m_{red} is the reduced mass of the atoms involved, ω the resonance angular frequency and r the distance between the atoms.

We have to take into account that our measured energy E_n is not the energy eigenvalue $E_{n,e}$ at the overtone but (since we measure the energy loss) the energy difference to the ground state energy $E_{0,e}$: $E_n = E_{n,e} - E_{0,e}$.

For an harmonic oscillator, the energy eigenvalues are equidistant since $E_{n,e} = \hbar\omega(n+\frac{1}{2})$. Here, $\hbar = \frac{h}{2\pi}$ is the reduced Planck constant. This gives for $E_{n,e}$:

$$E_{n,e} = E_n + E_{0,e} = E_n + \frac{1}{2n}E_n = E_n(1+\frac{1}{2n}) \quad [5]$$

Using the energy eigenvalues, ω can be written as

$$\omega = \frac{E_{n,e}}{\hbar(n+\frac{1}{2})} \quad [6]$$

Putting equation 6 into equation 4 as well as using equation 5 we obtain a harmonic potential $V_n(r)$ for each energy E_n:

$$\begin{aligned} V_n(r) &= \frac{1}{2}(\frac{E_{n,e}}{\hbar(n+\frac{1}{2})})^2 \cdot m_{red} \cdot r^2 \\ &= \frac{1}{2}\frac{E_n^2}{\hbar^2}\frac{(\frac{2n+1}{2n})^2}{(n+\frac{1}{2})^2} \cdot m_{red} \cdot r^2 \\ &= \frac{1}{2}\frac{E_n^2}{\hbar^2 n^2} \cdot m_{red} \cdot r^2 \end{aligned} \quad [7]$$

This method gives a set of harmonic potentials, one for each vibrational overtone n. For a reconstruction of a true harmonic potential the set of harmonic potential obtained would have the same shape, but since our measured energies do not follow a harmonic potential this will result in a set of gradually widening harmonic curves, each of which represents the PES width at a certain vibrational overtone.

We assume now that the width w_n of the nth harmonic potential at energy E_n corresponds to the width of the potential to be reconstructed at the energy E_n. The shape of the potential to be reconstructed for $R < R_{eq}$ is given by the gasphase potential from measurement (or alternatively from theory).

The width of the reconstructed potential is given by the width of the corresponding harmonic potential. To obtain the corresponding maximal bond elongation, we add the calculated harmonic potential width w_n to the value for the minimal bond elongation obtained from the gasphase potential at energy E_n. This gives a new poitn for the reconstructed potential at $R > R_{eq}$ for energy E_n, see Fig. S1. With all measured energies we are able to stepwise reconstruct the shape of the true potential energy surface.

For the isolated molecules in gasphase water with well separated partial density of the vibrational states of each overtone, the position of the fit intensity maximum is used as E_n. The resulting stepwise harmonic reconstruction is shown in Fig. S2 together with a number of gasphase ground state potentials obtained by variuous theoretical methods. All of these agree nicely with the stepwise reconstructed potential.

In the case of liquid water, the partial densities of the vibrational states overlap for higher overtones (1), thus making it possible to have more than one (i.e. 1 or 2) energy eigenvalue per experimental vibrational peak. For the stepwise harmonic reconstruction in this case, we have then to allow either one or two E_n per vibrational peak. A single E_n is handles analogous to the gasphase case; with 2 assumed energy eigenvalues per peak, the two energies at $\pm\frac{1}{2}$ FWHM intensity are chosen for E_{n1} and E_{n2}.

Validity of the stepwise harmonic reconstruction

To reconstruct a potential, different approaches and assumptions can be applied. One way that works very well for simple isolated molecules is the Morse reconstruction. However, the system studied might not be as simple and we also do not want to assume from the beginning that the potential has a Morse shape, i.e. an asymmetric potential with a dissociation limit. Within the stepwise harmonic reconstruction we assume that at every energy eigenvalue the potential behaves locally like a harmonic potential. This is analogous to the local density approximation (LDA) in DFT where the exchange correlation functional at a given point is assumed to only depend on the (local) electron density at that point.

Using a set of harmonic potentials for the reconstruction means naturally that we cannot totally reconstruct a Morse potential shape, but we can get fairly close to that without assuming that the system has a Morse potential from the beginning as it is shown for the $4a_1$ of gas phase water in Fig. S2. All theoretical and experimental curves show excellent agreement for $R \leq R_{eq}$ and only slight deviations for $R > R_{eq}$ that start for the DFT calculation at about $R - R_{eq} = 0.6$ Åand for the remaining potential curves at $R - R_{eq} > 1$ Å.

The stepwise harmonic reconstruction from Morse energies was used as a method to double check how much the systematic error affects a known potential shape. We start with a set of clean Morse potential energies (of the Morse reconstruction of gasphase water, black dotted line in Fig. S2) and use the stepwise harmonic reconstruction to extract the potential from those (light blue dotted line in Fig. S2). The potentials show very good agreement up to $R - R_{eq} = 1.5$ Åeven though the energy eigenvalues of the reconstructed potential differ from the original ones.

The question arises whether the symmetric and local asymmetric modes are strongly affected by the nuclear quantum effects in liquid water that systematically improve the agreement of first-principles simulations with experiment. Generally, quantum effects associated with the stretching motion of protons lead to a less structured liquid (2, 3). Our reconstruction scheme is based on a quantized coordinate, so in this sense the motion of the proton is considered at a quantum-mechanical level. Notwithstanding, we assume that the protons move along a one-dimensional coordinate, being affected by the immediate coordination environment. Because motion along other directions is neglected, it could mean that the steepness of our reconstructed potentials could be somewhat overestimated. However, the reconstructed potentials are meant to provide a measure of the distribution of local environments in liquid water, which is an overall larger effect than the correction due to the quantum motion of the protons.

Parameters of stepwise harmonic reconstruction for liquid water potentials shown in Fig. 4b and 5b

The correspondence of energy eigenvalues and vibrational peaks for the potentials obtained with the stepwise harmonic reconstruction for excitation at the liquid water $4a_1$ (shown in the figures 4b) and $2b_2$ (shown in figure 5b) are listed in tables S1 and S2, respectively.

Potential energy surfaces of liquid water on the $4a_1$ and $2b_2$

Potentials along the asymmetric O-H bond coordinate: Ab initio simulations show that apart from the first vibrational peak, all further peaks can contain 1 or 2 energy eigenvalues (1). Applying this for the harmonic reconstruction together with the measured vibrational progression of the elastic peak and vibrational overtones for excitation at the $4a_1$ in liquid water, we obtain a set of permutations of 1 and 2 energy eigenvalues that allows to reconstruct the full set of possible potential energy surfaces along the asymmetric OH bond coordinate in the liquid water molecule, see Fig. S3 in comparison to the Morse reconstructed potential and the ab inito calculations from (1).

Fig. S4 shows the corresponding potentials along the symmetric normal mode, excited at the water $2b_2$ from stepwise harmonic reconstruction.

vibrational peak	eigenvalues per peak					
	red	blue	yellow	green	pink	lavender
1	1	1	1	1	1	1
2	1	2	2	2	2	1
3	1	2	1	2	1	1
4	1	2	1	2	2	1
5	1	2	1	2	1	1
6	1	2	1	1	2	2
7	1	2	1	1	1	2
8	1	2	1	1	2	2
9	1	2	1	1	1	2

Table S1. Number of energy eigenvalues per vibrational peak for the reconstructed potentials at the $4a_1$ resonance of liquid water. The different colors correspond to the colors of the different potentials shown in figure 4b.

	eigenvalues per peak					
vibrational peak	red	blue	yellow	green	pink	lavender
1	1	1	1	1	1	1
2	1	2	2	2	2	1
3	1	2	1	2	1	1
4	1	2	1	1	2	2
5	1	2	1	1	1	2

Table S2. Number of energy eigenvalues per vibrational peak for the reconstructed potentials at the $2b_2$ resonance of liquid water. The different colors correspond to the colors of the different potentials shown in figure 5b.

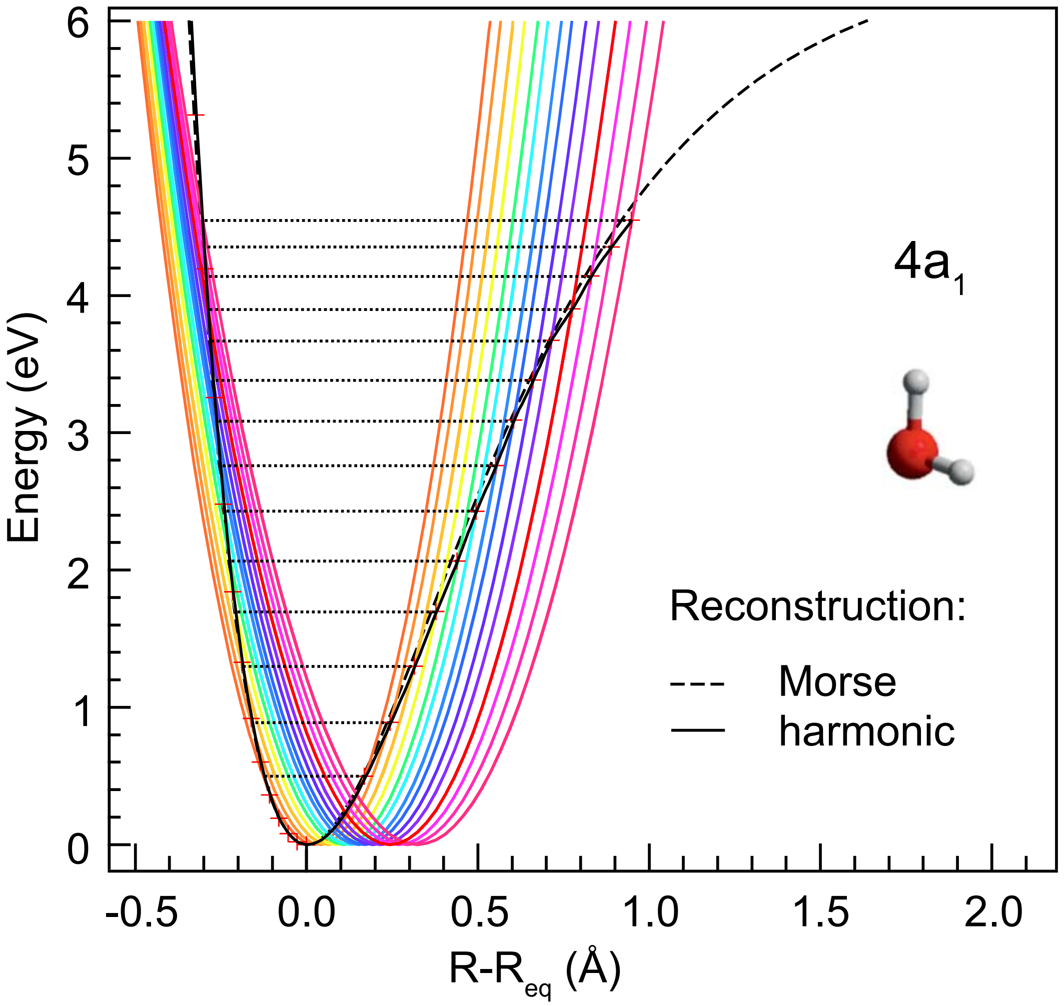

Fig. S1. Harmonic reconstruction of a potential energy surface from vibrational overtone energies measured with RIXS and comparison to the Morse reconstruction.

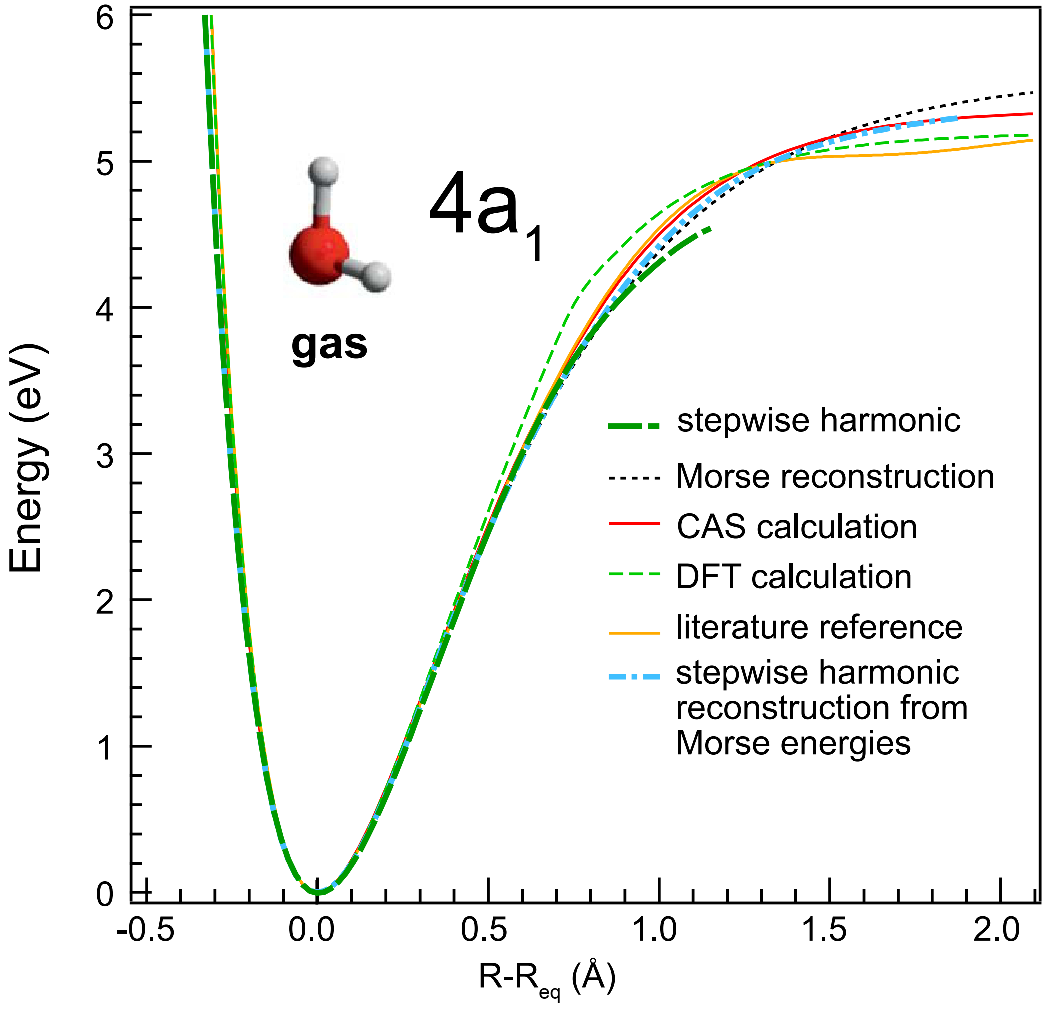

Fig. S2. Comparison of the potential energy surface of gasphase water for excitation on the 4a1 obtained by several experimental and theoretical methods as well as a literature reference (4). While the part for $R > R_{eq}$ differs, the part for $R < R_{eq}$ remains very similar. See for numerical values of the stepwise harmonic reconstructed potential the Dataset 1, for the Morse reconstruction the Dataset 2, and for the DFT calculation the Dataset 3 in the supplementary material. The CAS calculation is taken from reference (5).

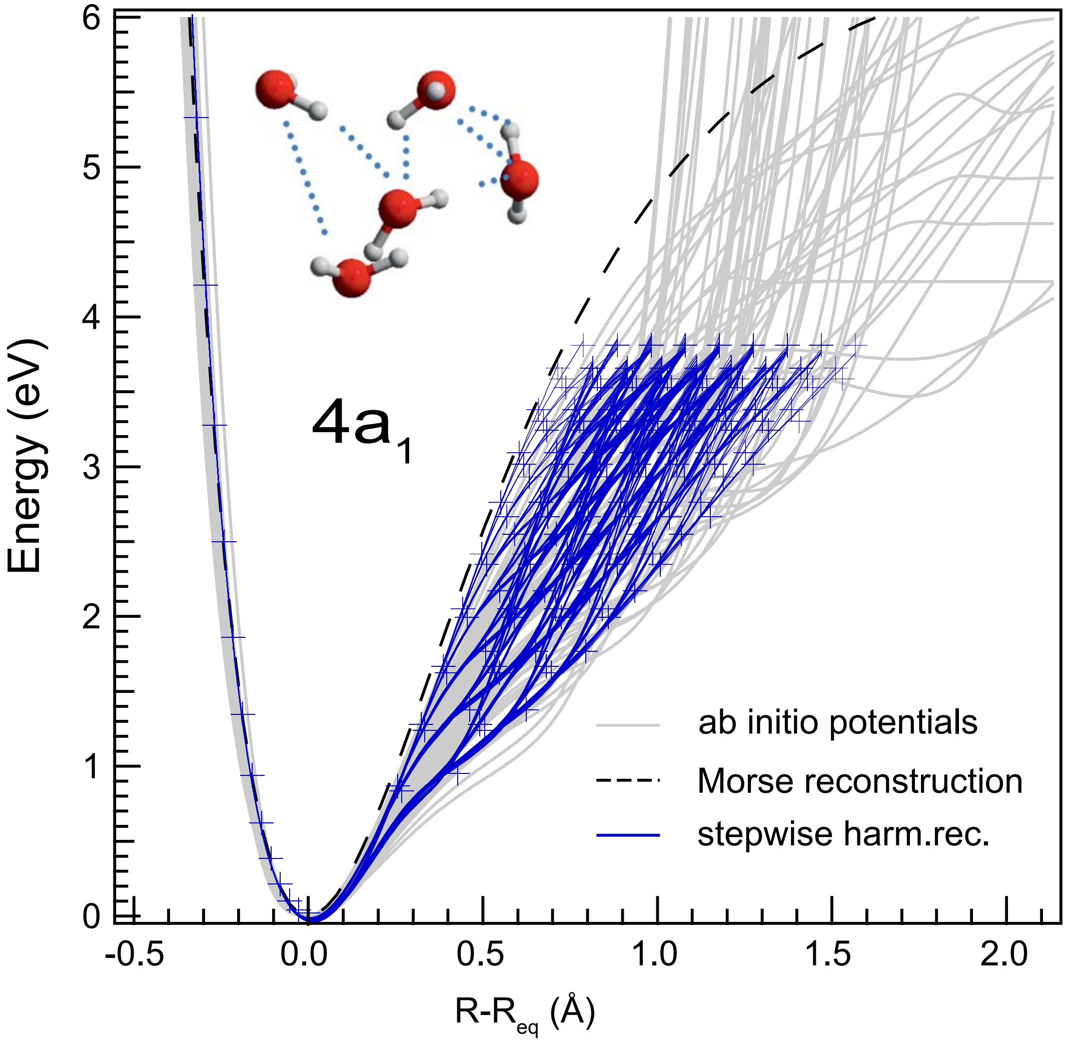

Fig. S3. Harmonic reconstruction of all possible potential energy surfaces along the asymmetric OH bond coordinate in liquid water in comparison to Morse reconstruction of the potential and ab inito calculations. See for numerical values of the stepwise harmonic reconstructed potentials the Dataset 4, for the Morse reconstruction the Dataset 5 in the supplementary material. The ab initio potentials (in grey) are taken from reference (1).

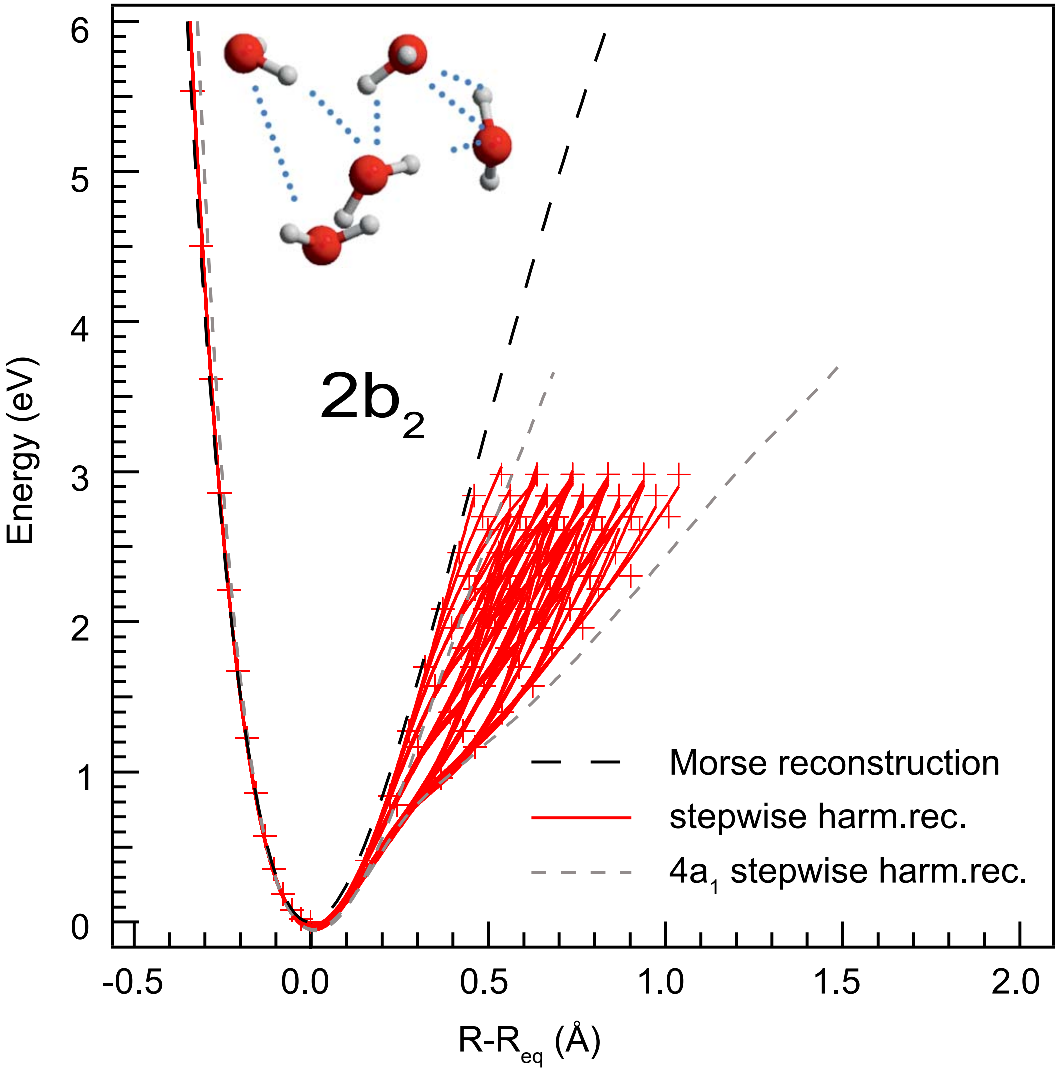

Fig. S4. Harmonic reconstruction of all possible potential energy surfaces along the diagonal between the OH bonds in liquid water (excitation of the symmetric normal mode) in comparison to Morse reconstruction of the potential. For comparison, the steepest and shallowest potentials along the OH bond from Fig. S3 are marked in grey. See for numerical values of the stepwise harmonic reconstructed potentials the Dataset 6 and for the Morse reconstruction the Dataset 7 in the supplementary material.

References

1. Vaz da Cruz V, et al. (2019) Probing hydrogen bond strength in liquid water by resonant inelastic x-ray scattering. *Nature Commun.* 10:1013.
2. Morone JA, Car R (2008) Nuclear quantum effects in water. *Phys. Rev. Lett.* 101:017801.
3. Ceriotti M, et al. (2016) Nuclear quantum effects in water and aqueous systems: Experiment, theory, and current challenges. *Chem. Rev.* 116:7529.
4. Patridge H, Schwenke DW (1997) The determination of an accurate isotope dependent potential energy surface for water from extensive ab initio calculations and experimental data. *J. Chem. Phys.* 106:4618.
5. Eckert S, et al. (2018) One-dimensional cuts through multidimensional potential-energy surfaces by tunable x rays. *Phys. Rev. A* 97:053410.
